# Cross-cultural adaptation and validation of the 15-item Geriatric Depression Scale (GDS-15) into Igbo language: a validation study

**DOI:** 10.1186/s12955-022-01928-8

**Published:** 2022-02-05

**Authors:** Ukamaka Gloria Mgbeojedo, Christopher Olusanjo Akosile, Juliet Chidera Ezugwu, Emmanuel Chiebuka Okoye, Jeneviv Nene John, Kenneth Umezulike Ani, Obinna Chinedu Okezue

**Affiliations:** 1 Department of Medical Rehabilitation, College of Medicine, University of Nigeria, Enugu Campus, Nsukka, Enugu Nigeria; 2 Department of Medical Rehabilitation, College of Health Sciences, Nnamdi Azikiwe University, Nnewi Campus, Awka, Anambra Nigeria

**Keywords:** Geriatric Depression Scale, Cross-cultural adaptation, Validation, Igbo culture

## Abstract

**Background:**

Late-life experiences such as protracted and indisposing medical disorders can negatively impact older adults’ psychological and mental health, making them vulnerable to depression. Majority of the assessment tools for depression were developed for use in western countries. There is therefore the need for availability of culture- and environment-specific tools for assessment of depression in low-and-middle-income countries. This study was designed to cross-culturally adapt and validate the Geriatric Depression Scale-15 (GDS-15) into Igbo language and culture.

**Methods:**

The English version of the GDS-15 was translated into Igbo language; synthesized, back-translated, and underwent expert panel review, pretesting and cognitive debriefing interview, according to the American Academy of Orthopedic Surgeons’ guidelines. The Igbo version of the GDS-15 was tested for concurrent and structural validities, and internal consistency among consecutively recruited 140 consenting older adults (62.9% females) in Enugu North Senatorial District at 0.05 level of significance.

**Results:**

The English version of the GDS-15 was successfully cross-culturally adapted to Igbo with all the 15 items still retained on the Igbo version of the GDS-15. The Igbo version of the GDS-15 exhibited the same structure as the English version, and displayed a Cronbach’s alpha value of 0.53 with no significant ceiling (0%) and floor (0%) effects. The correlation between the participants’ total scores on the Igbo and the English versions of the GDS-15 (ρ = 0.86) was adequate. There was no significant difference between corresponding scores in the English and Igbo versions of the GDS-15 (*p* = 0.89).

**Conclusions:**

The Igbo version of the GDS-15 is a valid and culturally specific instrument, and can be used for assessing depression among Igbo older adults in Nigeria.

## Introduction

Globally, there is a rapid increase in the number of people aged 60 years and above [1, 2]. In most of the developed and developing countries, the age of 60–65 is considered equivalent to retirement age, and it is said to be the beginning of old age [3]. About 3.4% or 5.9 million of the total Nigerian population are older adults (aged 65 years and above) [4]. Though ageing population is a sign of victory of human development [5], it comes with challenges like depression in the society [1].

Depression is a common psychological illness characterized by persistent sadness and a loss of enthusiasm in activities that individuals usually enjoy, accompanied by an inability to accomplish daily activities [6]. It might be influenced by incidental and unplanned negative life events, including: death of loved ones, elderly abuse, physical and cognitive decline, retirement, restricted mobility, limited financial resources, seclusion, and an inability to participate in once-cherished activities, and so on [7–10]. Common indications of depression among older adults include feelings of hopelessness, helplessness, weight changes, persistent sadness, and unnecessary stresses over funds, and so on. It often spurs thoughts of suicide and suicidal mortality in this older population [11]. Individuals who are single, unmarried, separated or widowed, and female are at greater risk of having depressive symptoms [8, 12].

Globally, the prevalence of depression has been reported to be 4.4% [13, 14]. However, different prevalence rates of depressive symptoms have been reported among different populations: 41.1% for a Sudanese population [15], 14.5% for an African American population [16]; 39.6% for a South-African population [17], 12% for a Greek population [18], 14.4% for an Indian population [19], 23.7% and 41.8% for Mexican and Ethiopian population respectively [8, 20]. In Nigeria, the prevalence of depression among older adults in South-southern, South-western and South-eastern populations has been reported to be 49.5%, 21.4% and 19% respectively [16, 21, 22]. However, late-life depression, in spite of its prevalence and effect, may be difficult to diagnose and insufficiently treated [23, 24]. Active screening, and early detection and treatment may be effective in reducing healthcare cost and improving case finding for depression among older adults [25, 26]. Therefore, there is need for availability of validated instruments for assessing depression [17, 26].

Many of the measurement tools used to assess depression among older adults are adaptations of instruments developed in other cultural settings [27]. This includes the EURO-D, the Beck Depression Inventory, the Geriatric Depression Scale (GDS), the Center for Epidemiological Studies Depression Scale, and the Zung Self-Rating Depression Scale [26]. The most commonly used depression screening tools for older adults are the GDS and the Center for Epidemiological Studies Depression Scale [17]. The original GDS is a psychometrically-sound 30-item simple-to-administer questionnaire that has been utilized generally among community and institutionalized older adults [28, 29]. Sheikh and Yesavage [30] developed and validated a 15-item GDS Short Form so as to improve its acceptability for the older adult population and make this measure an easier screening tool for depression.

Diagnostic scales developed in the western world may be inappropriate for use in developing countries. Furthermore, the possibility of cultural effects confounding the measurement of depression may influence the individual responses to questionnaires, and may therefore prompt misdiagnosis or improper assessment of depression [26, 31, 32]. It is therefore advisable and pertinent to cross-culturally adapt an instrument before use in a different culture and setting [33]. Cross-cultural adaptation of an already existing instrument instead of developing a new one has often been the choice when addressing a population whose language is different from the source language, as it is more economical and ensures ease of comparisons among populations [33–36]. Cross-cultural adaptation comprises the translation, adaptation and validation phases.

According to the National Bureau of Statistics [37], the Nigerian adult literacy in English Language is 42.1%. With this literacy level, it is imperative to make provision for a validated instrument for the measurement of depression especially among Nigerian older adults who are not fluent in or understand English language. Mere translating the tool to this group of participants by different assessors will introduce some level of biases as the translations are not validated and may differ significantly with individuals. The GDS-15 has been utilized in various languages and cultural settings to assess depression, and has proven to be a reliable and valid instrument [29, 38, 39]. The GDS-15 has been translated into various languages: Iranian [40], Japanese [41], Malay [42], Chinese [43], Spanish [44], and Nepalian [38]. However, there is no Nigerian adapted and validated version of the GDS-15. This study was therefore designed to translate, culturally adapt and validate the English version of the short form of GDS-15 into Igbo language. Igbo language is one of the three major Nigerian native languages (making up about 18% of the whole Nigerian population) and a minor language in Equatorial Guinea, with over 24 million speakers [45–47].

## Methods

### Design

This was a validation study that utilized the recommendations for cross-cultural adaptation established by Beaton et al. [33] for the American Academy of Orthopedic Surgeons. The study protocol was approved by the Ethical Review Committee of the University of Nigeria Teaching Hospital, Ituku-Ozalla, Enugu (NHREC/05/01/2008B-FWA00002458-1RB00002323). Permission to translate and validate the original English version of the GDS-15 was gotten from the developers. Informed consent was obtained from all the consecutively recruited older adult participants (65 years and above) in Enugu North Senatorial District, Enugu State, Nigeria. The purpose and procedures of the study were thoroughly explained to the respondents. Participants were assured of confidentiality and the freedom to withdraw from the study when they were no longer interested. The participants were older adults (≥ 65 years) who could understand both English and Igbo languages; were well-oriented in time, place and person; and were not on any antidepressants. These eligibility criteria were for both the adaptation and validation phases of the questionnaire. Information on participants’ socio-demographic variables (age, gender, occupation, level of education, and marital status) was obtained through oral interview.

### Instruments for data collection

#### The Geriatric Depression Scale (GDS)

The GDS is used in the assessment of depression among older adults. The GDS, first created by Sheikh and Yesavage [30] has been tested and used extensively with the older population. It is a time-saving instrument with a response format of (Yes/No), which brought about its use in different healthcare settings [48]. The long form of GDS is a brief, 30-item questionnaire in which participants are asked to respond by answering yes or no in reference to how they felt over the past week. Questions from the long form of GDS which had the highest correlation with depressive symptoms in validation studies were selected for a shorter version (GDS-15) that has 15 items. A scoring grid accompanies the GDS-15:

**Table:** 

1) NO	2) YES	3) YES	4) YES	5) NO	6) YES	7) NO	8) YES
9) YES	10) YES	11) NO	12) YES	13) NO	14) YES	15) YES	

Each question is scored as either 0 or 1 point. One point is given for each respondent’s answer that matches those on the grid. For example, the grid response to “are you basically satisfied with your life?” is ‘NO’. If the elderly person responds in the negative, one point is scored; if the response is ‘YES’, then no point is scored. Of the 15 items, 10 indicated the presence of depression when answered positively, while the rest (question numbers 1, 5, 7, 11, 13) indicated depression when answered negatively. Scores of 0–4 are considered normal; 5–8 indicate mild depression; 9–11 indicate moderate depression; and 12–15 indicate severe depression. The GDS was found to have 92% sensitivity and 89% specificity when evaluated against diagnostic criteria. The validity and reliability of the tool have been supported through both clinical practice and research. The GDS-15 has a moderate internal consistency (Cronbach’s α = 0.82) [30].

#### Cross-cultural adaptation and validation of the GDS-15

This study followed the American Academy of Orthopaedic Surgeons’ guidelines for cross-cultural adaptation developed by Beaton et al. [33]. The procedure for the study was performed in sequential order: phases 1(translation), 2 (adaptation) and 3 (validation).

#### Phase 1: translation phase

This stage involves forward translation, synthesis of the translation and back translation [33, 34]. The forward translation involved the translation of the English version of the GDS-15 into Igbo language. Two bilingual translators (a linguist and a physiotherapist), whose mother tongue was Igbo language, independently translated the questionnaire to produce two different Igbo translations (FT1 and FT2). The physiotherapist was aware of the concepts of the questionnaire being translated. The purpose of this translation (by the physiotherapist) was to offer equivalence from a more clinical perspective and also to produce a translation providing a more reliable equivalence from a measurement perspective. The linguist had no medical or clinical background, and was neither aware nor informed about the concepts being quantified. This second translator was supposed to provide a language translation as used by the population, in order to accentuate obscure meanings in the original questionnaire [46].

The two translations were then harmonized and synthesized by the two translators to produce a single quality-assured version (ST-12). The ST-12 was translated back to English language (as a quality control step) by two different bilingual translators who were oblivious of the concept under study, producing two back-translated English versions BT1 and BT2. These translators were physiotherapy lecturers who were fluent in both English and Igbo languages, and also knowledgeable in cross-cultural adaptation procedure. The essence of this procedure was to ascertain the validity of the questionnaire in order to ensure that the translated version reproduced similar item content as the original version [34].

#### Phase 2: adaptation phase

The original English GDS-15 version (E-GDS), the two forward translations, the synthesized version, and the back translations underwent an expert panel review in order to produce a version expertly cross-culturally adapted to Igbo language, culture and environment. The expert panel comprised the four translators, four physiotherapy researchers, and a lay person. The expert committee were very familiar with the Igbo culture and environment, and identified the comprehensiveness, relevance and comprehensibility of the instructions, items and response options. All the translations were carefully deliberated on while ensuring experiential, operational, measurement, conceptual, idiomatic, and semantic equivalences. All disagreements in the translations were settled by consensus, thus producing a pre-final Igbo version of the questionnaire which was subjected to field testing by administering it to 30 older adults (62.9% female) who were consecutively recruited from conveniently selected communities in Enugu North Senatorial District after informed consent had been sought and obtained from them. These participants were taken through cognitive debriefing interview in order to ascertain: what each participant thought was meant by each item on the questionnaire and the responses; ease of understanding each item; if there was ambiguity in each item; if the response items for the items were difficult to understand; and if the activity described in each item was being experienced in Igbo culture. Each participant was expected to answer ‘YES’ or ‘NO’ for each question.

The answers on the cognitive debriefing checklist were then presented to the expert panel on a second meeting. Items or response option with less than 80% ‘YES’ were supposed to be amenable to changes. Item 4 “Ọ bụ na  ị dịghị enwekarị mmụọ mkpali?” scored 60%. This item was misunderstood/misconstrued by the participants. It was therefore amended to “ị naghị enwe uchu ịme ịhe ọ bụla?”. However, the remaining items had at least 80% ‘YES’. No further modification was made on the questionnaire by the panel of experts. The final Igbo version of the GDS-15 (I-GDS-15) was consequently produced.

#### Phase 3: validation phase

This was the final stage, and it involved testing the psychometric properties of the instrument (I-GDS-15). The E-GDS-15 and the I-GDS-15 were either self-administered or researcher-administered based on each participant’s preference to 140 older adults who met the inclusion criteria. The E-GDS-15 was administered in order to determine the concurrent validity of the I-GDS-15. The order of the administration of the two versions of the questionnaire was randomized using simple randomization method. Participants who picked ‘E’ answered the E-GDS-15 first while those who picked ‘I’ answered the I-GDS-15 first. A sample size of 140 had a 94% power to detect a medium change of 0.3 at an alpha level of significance of 0.05. Sample size was estimated using G* Power 3.0.10 [49].

### Data analysis

Obtained data were analyzed using the Statistical Package for Social Sciences (SPSS) version 21 (IBM Corp., New York, USA). The demographic profile of the participants and the scores from the two versions of the questionnaire were summarised using frequency counts, percentages, median, range, mean and standard deviation. The Spearman rank order correlation and scatter plot were respectively used to estimate and pictorially show the level of correlation between participants’ scores on the I-GDS-15 and the E-GDS-15, in order to ascertain the concurrent validity of the I-GDS-15. The questionnaire was tested for ceiling and floor effects. The questionnaire would be regarded as having ceiling or floor effect if more than 15% of the participants achieved maximum or minimum possible scores respectively [50]. The Cronbach’s alpha was used to ascertain the internal consistency of the I-GDS-15. Bland–Altman plot was used to depict the homoscedasticity of the total scores on the I-GDS-15 and the E-GDS-15. Principal component analysis (PCA) was used to estimate the structural validity of the I-GDS-15. PCA is a technique for reducing the dimensionality of datasets, increasing interpretability, while still retaining the original information. Standard error of mean (SEM) and the minimal detectable difference (MDD) of the total and item scores on the I-GDS were determined. The MDD was estimated using the following formula: MDD = 1.96 × SEM × √2 [51].

Before performing the PCA, the Barlett’s test of sphericity and the Kaiser–Meyer–Olkin (KMO) were used to check the appropriateness of the data for factorial analysis. To ensure the factorability of the data, Barlett’s test of sphericity value must be significant [52], and the KMO value must exceed the recommended value of 0.6 [53]. Furthermore, all the coefficients of correlation of each of the items on the I-GDS-15 with one another must all exceed 0.3; and the communalities must all be above 0.3 [54, 55]. Communality values of less than 0.3 might suggest that the item did not fit well with the other items loading on the same component. During PCA, any factor with its Eigen values exceeding 1 are often retained. The number of retainable factors could also be revealed through the scree plot by counting off the number of points before a clear point of reflection [56]. Every component with initial Eigen value lower than the random Eigen value are usually rejected. Level of significance was set at *p* < 0.05.

## Results

### Cross-cultural adaptation of the GDS-15 into Igbo

The pre-final version of the I-GDS-15 was pretested on 30 older adults who were taken through cognitive debriefing interview. Some modifications were made on four of the items during the expert panel meeting. The word “basically” in item 1 was deliberated on for the best Igbo synonym that captured the context. “Ozuzu oke” was contemplated on but was dropped by the panel as it was thought not to convey the in-depth meaning of “basically”. After deliberations on several likely alternative/synonym for it, the panel opted for “o pekata mpe”. In item 3, the word “empty” was taken to mean “void”. The initial Igbo translation “abaghịzi uru” was argued to mean “useless”. Studying one of the forward translations, the panel agreed with the translation “chakoo” to mean “emptiness”. “Good spirits” in item 5 was initially translated to be aṅụrị/obi ọma. Being in good spirits and being happy were argued to mean the same thing. While some argued that good spirits could mean cheerful (obi ọma/aṅụrị), others disagreed. After many considerations, it was literally translated to “ezi mmụọ”. However, it was argued that it may seem offensive to some individuals on cultural/belief basis. The panel therefore deliberated further for a more polite appropriate and acceptable word. “Obi sara sara” was finally agreed to be a better option that conveyed the meaning. The word “helpless” in item 8 was also literally translated to “enweghị enyemaka”. This was argued not to represent the contextual meaning. The panel agreed that it did not mean “being without assistance/aid”. The word “helpless” was highlighted as being a dead-end situation that could be salvaged only by a miracle. After several disagreements, the panel opted for “nkoropu obi”.

However, during the pretesting of the pre-final I-GDS-15, all the participants indicated clarity of language and ease of understanding of all the items during cognitive debriefing interview except for two items, and so led to some modifications at the second expert meeting. The term “mmụọ mkpali” in item 4 was misunderstood to be “insulting”. It was modified to “Ị naghị enwe uchu ịme ihe ọ bụla?” In item 13, "ume" and "ike" were thought to mean similar things. The expert review opted for “ike”. No further modifications were made, and thus the final I-GDS-15 was produced. Table 1 summarises the modifications (final I-GDS-15).

**Table 1:** Socio-demographic profiles of study participants

Parameters	Class	Frequency	Percentage (%)
Age	65–70	95	67.9
	70–80	32	22.9
	80–90	9	6.4
	90–100	4	2.9
Gender	Male	52	37.1
	Female	88	62.9
Marital status	Single	1	0.7
	Married	96	68.6
	Widowed	42	30
	Divorced	1	0.7
Occupation	Unemployed	17	12.1
	Farming	24	17.1
	Civil/public service	33	23.7
	Retirees	40	28.6
	Artisans	4	2.8
	Trading	22	15.7
Educational attainment	No formal education	46	32.9
	Primary	33	23.6
	Secondary	38	27.1
	Tertiary	23	16.4

### Validation of the Igbo version of the GDS-15

#### Socio-demographic profile of the participants

One hundred and forty (140) apparently healthy older adults (37.1% males; 67.9% falling within 65–70 years old) who were residents in Enugu North Senatorial District were involved in the psychometric testing of the I-GDS-15. The description of the participants is displayed on Table 1.

#### Concurrent validity of the Igbo version of the GDS-15

**Fig. 1:**
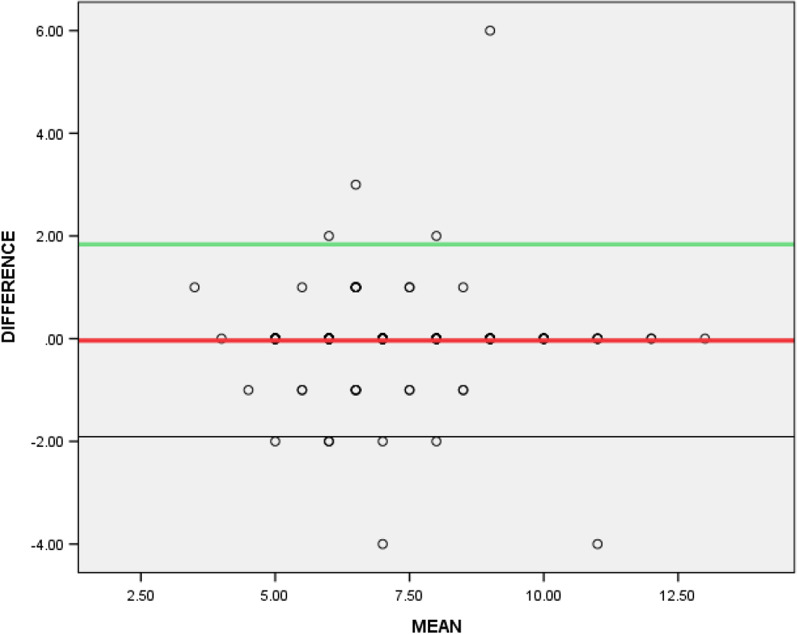
Bland–Altman plot of the total scores on the I-GDS-15 and the E-GDS-15

**Fig. 2:**
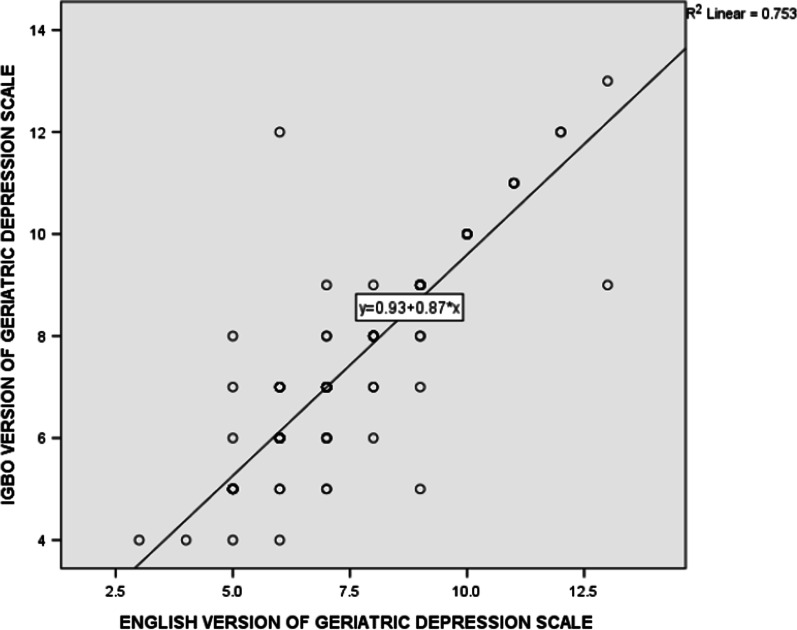
Scatter diagram for total scores on the I-GDS-15 and the E-GDS-15

Spearman correlation coefficients of the item and total scores ranged from poor to excellent (0.271–1.000), with items 14 and 11 having the highest (*ρ* = 1.000; *p* < 0.001) and the lowest (*ρ* = 0.271; *p* = 0.001) correlation coefficients respectively (Table 2). Total score on the E-GDS-15 and the I-GDS-15 as pictorially shown on the Bland–Altman plot revealed evidence of homoscedasticity of the two scores (Fig. 1). The Scatter diagram demonstrates the correlation between the total scores on the E-GDS-15 and the I-GDS-15 (Fig. 2).The Mann–Whitney U test showed no significant difference between the total scores on the two versions of the questionnaire (U = 9710.500; *p* = 0.89), thus revealing that the two instruments produced equivalent conceptual and linguistic meanings (Table 3). The ceiling and floor effects are 0% respectively as no participant scored the lowest or the highest score.

**Table 2:** Spearman rank order correlation between the items in the E-GDS-15 and the I-GDS-15

Variables	ρ	*p*	Rating
GDS1	0.793	0.000	Adequate
GDS2	0.762	0.000	Adequate
GDS3	0.952	0.000	Adequate
GDS4	0.673	0.000	Adequate
GDS5	0.529	0.000	Adequate
GDS6	0.868	0.000	Adequate
GDS7	0.888	0.000	Adequate
GDS8	0.778	0.000	Adequate
GDS9	0.904	0.000	Adequate
GDS10	0.851	0.000	Adequate
GDS11	0.271	0.001	Poor
GDS12	0.878	0.000	Adequate
GDS13	0.736	0.000	Adequate
GDS14	1.000	0.000	Adequate
GDS15	0.937	0.000	Adequate
Total	0.860	0.000	Adequate

E-GDS-15: English version of the Geriatric Depression Scale questionnaire

I-GDS-15: Igbo version of the Geriatric Depression Scale questionnaire

**Table 3:** Mann Whitney U test comparing scores on the E-GDS-15 and the I-GDS-15

Variable	Mean ± SD		U	P
	English	Igbo		
GDS1	0.96 ± 0.19	0.96 ± 0.19	9800.000	1.000
GDS2	0.84 ± 0.37	0.84 ± 0.37	9730.000	0.871
GDS3	0.17 ± 0.39	0.19 ± 0.39	9660.000	0.755
GDS4	0.24 ± 0.43	0.25 ± 0.44	9730.000	0.890
GDS5	0.93 ± 0.26	0.94 ± 0.23	9660.000	0.627
GDS6	0.10 ± 0.30	0.13 ± 0.34	9520.000	0.453
GDS7	0.93 ± 0.26	0.94 ± 0.23	9660.000	0.627
GDS8	0.38 ± 0.49	0.31 ± 0.47	9170.000	0.259
GDS9	0.16 ± 0.37	0.19 ± 0.39	9520.000	0.527
GDS10	0.40 ± 0.49	0.40 ± 0.49	9800.000	1.000
GDS11	0.97 ± 0.17	0.98 ± 0.15	9730.000	0.702
GDS12	0.09 ± 0.29	0.10 ± 0.30	9730.000	0.840
GDS13	0.82 ± 0.38	0.74 ± 0.44	8960.000	0.085
GDS14	0.08 ± 0.27	0.08 ± 0.27	9800.000	1.000
GDS15	0.16 ± 0.37	0.15 ± 0.36	9660.000	0.743
Total	7.24 ± 1.86	7.20 ± 1.86	9710.500	0.893

E-GDS-15: English version of the Geriatric Depression Scale questionnaire

I-GDS-15: Igbo version of the Geriatric Depression Scale questionnaire

### Reliability analysis

Cronbach’s alpha for the internal consistency coefficient of all the items on the I-GDS-15 was 0.53. This value suggests poor internal consistency of the items on the I-GDS-15. The standard error of mean and minimum detectable difference values for the total and item scores on the I-GDS ranged from 0.01 (item 11) to 0.16 (total score) and 0.03 to 0.44 respectively (Table 4). This revealed evidence of the responsiveness of the item and total scores on the scale.

**Table 4:** Standard error of mean and minimal detectable difference of the item and total scores on the I-GDS

Items	SEM	MDD
I-GDS1	0.02	0.06
I-GDS2	0.03	0.08
I-GDS3	0.03	0.08
I-GDS4	0.04	0.11
I-GDS5	0.02	0.06
I-GDS6	0.03	0.08
I-GDS7	0.02	0.06
I-GDS8	0.04	0.11
I-GDS9	0.03	0.08
I-GDS10	0.04	0.11
I-GDS11	0.01	0.03
I-GDS12	0.03	0.08
I-GDS13	0.04	0.11
I-GDS14	0.02	0.06
I-GDS15	0.03	0.08
Total	0.16	0.44

SEM: Standard error of mean; MDD: minimal detectable difference

### Structural validity of the Igbo version of the GDS-15

The Kaiser–Meyer–Olkin (KMO) measure of sampling adequacy was 0.67 exceeding the recommended value of 0.6 [52], while the Barlett’s test of sphericity was statistically significant (X^2^ = 306.352; *p* < 0.001); thus suggesting that factor analysis was appropriate and feasible.

**Fig. 3:**
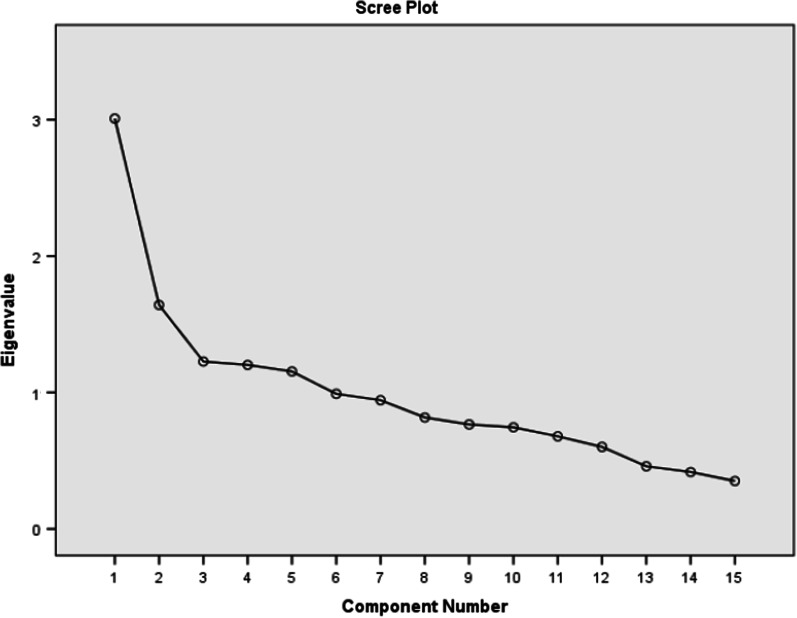
Scree plot of the components on the Igbo version of the Geriatric Depression Scale-15

The analysis revealed that there were five factors with their eigenvalues greater than 1, accounting for 20.05, 10.94, 8.18, 8.01 and 7.69 of the variances respectively. The five components thus explained a total of 54.87% of the variances (Table 5). Scree plot also depicted the presence of these five factors (Fig. 3). The communality values of all the items were all above 0.3 indicating that the items fit well with one another (Table 6).

**Table 5:** Principal Component Analysis and Monte Carlo parallel analysis of the I-GDS-15

Component	Initial Eigen values	Decision	Variances%	Cumulative%
C1	3.008	Accepted	20.054	20.054
C2	1.641	Accepted	10.943	30.997
C3	1.227	Accepted	8.180	39.177
C4	1.202	Accepted	8.014	47.191
C5	1.154	Accepted	7.693	54.883
C6	0.990	Rejected	6.600	61.484
C7	0.944	Rejected	6.290	67.774
C8	0.817	Rejected	5.445	73.219
C9	0.766	Rejected	5.104	78.323
C10	0.745	Rejected	4.967	83.290
C11	0.678	Rejected	4.523	87.813
C12	0.601	Rejected	4.006	91.819
C13	0.459	Rejected	3.060	94.880
C14	0.417	Rejected	2.779	97.659
C15	0.351	Rejected	2.341	100.000

I-GDS-15: Igbo version of the Geriatric depression scale

**Table 6:** Communalities of the items on the I-GDS-15

Items	Initial	Extraction
I-GDS1	1.000	0.640
I-GDS2	1.000	0.329
I-GDS3	1.000	0.614
I-GDS4	1.000	0.462
I-GDS5	1.000	0.717
I-GDS6	1.000	0.404
I-GDS7	1.000	0.563
I-GDS8	1.000	0.510
I-GDS9	1.000	0.626
I-GDS10	1.000	0.510
I-GDS11	1.000	0.559
I-GDS12	1.000	0.588
I-GDS13	1.000	0.539
I-GDS14	1.000	0.602
I-GDS15	1.000	0.571

Extraction Method Principal Component Analysis, I-GDS-15: Igbo version of the GDS-15

## Discussion

This study was performed to cross-culturally adapt and validate the Igbo version of the GDS-15 among Igbo older adults in Enugu, Southeast Nigeria following an established and accepted protocol [33] in order to minimize bias and eliminate erroneous results. The GDS-15 has been translated into various languages: Iranian [40], Japanese [41], Malay [42], Chinese [43], Spanish [44], and Nepalian [38].

There was significant correlation between the participants’ total scores on the I-GDS-15 and the E-GDS-15 (ρ = 0.86; *p* < 0.01), revealing an adequate concurrent (known-group) validity of the I-GDS-15. This indicates that the translated version is contextually equivalent to the original instrument. Hence, the I-GDS-15 may be used among Igbo-speaking individuals irrespective of their location with the probability of achieving same results. This was reiterated by the finding of no significant difference between the total scores on the E-GDS-15 and the I-GDS-15.

No participant had either the highest or the lowest total scores on the I-GDS-15, indicating that the scale had no ceiling nor floor effects. This shows that the scale can differentiate individuals at the same tail end of the scoring spectrum. The Cronbach’s alpha of the I-GDS-15 was 0.53, thus suggesting a poor internal consistency. This value is lower than the 0.82 Cronbach’s alpha of the original version [30]; and those reported in the Iranian: 0.92 [40]; Nepalian: 0.79 [38]; Japanese: 0.83 [41]; Malay: 0.75 [42] versions of the GDS-15. This finding may suggest that the items on the Igbo version of the Geriatric Depression Scale are heterogeneous. Depression is a subjective psychological diverse variable which measurement requires the exposure of individual emotions and feelings. Hence, responses to the items may differ, thus resulting in poor internal consistency coefficient. However, it should be noted that a too-high alpha value may suggest that some items are redundant as they are testing the same question but in a different guise [57]. A high reliability coefficient does not usually ensure accuracy of the study, rather it provides basis for making inferences about changes [58]. Streiner [59] recommended a maximum alpha value of 0.90. The MDD values of the I-GDS were determined in this study. This may be of help in the future in knowing whether an intervention study has produced a significant change in depression among older adults.

The participants’ data on the I-GDS-15 were subjected to factorial analysis (which is usually involved with the question of validity) because the Kaiser–Meyer–Olkin value (0.67) exceeded the recommended value of 0.6 [53]. Principal component analysis was selected instead of exploratory factor analysis because the scale had already been established on an existing theory by the original authors of the instrument [60]. Also, the communality values of all the items were all above 0.3 indicating that all the items fit well with other items. Furthermore, the Barlett’s test of sphericity also revealed evidence of significant correlation in the random matrix. However, the correlation coefficients of many of the items on the I-GDS with one another did not exceed the recommended value of 0.3. This further explains the not-so-high internal consistency coefficient (0.53) reported in this study. The analysis of this study revealed that there were five factors with their eigenvalues greater than 1, thus suggesting a five-factor structure. In a previous confirmatory factor analysis study, a five-factor structure was also revealed [61]. However, the factor structure of the GDS-15 still lacks consensus in literature; and varies across different language versions and cultures [62]. This therefore implies that the factor scores of the GDS should be interpreted with caution especially when it is administered in languages other than English.

## Limitations

The present study has some limitations. Individuals who could not understand English language were not involved in the study, which might have introduced some level of bias. However, this was a prerequisite of the chosen protocol. Also, the participants in this study were recruited from only a few communities in Enugu North Senatorial District thereby leaving out other communities with varying Igbo accents. However, the use of the central Igbo dialect commonly understood by every Igbo-speaking individual was reasoned to have addressed this recruitment bias; thus, ensuring generalizability of this study to all Igbo speakers across the globe. We make recommendations for further studies to explore the test–retest reliability and the clinimetric properties (of the I-GDS-15), which usually represent a step forward to the validation process of rating scales to be used in clinical research and practice [63].

## Conclusions

The I-GDS-15 is a valid and an acceptable instrument for use in measuring depression among Igbo-speaking older adults. It is recommended that the GDS-15 be translated, adapted and validated to other major Nigerian languages and cultures. Further studies should be conducted to determine other psychometric and clinimetric properties of the I-GDS-15.

## Data Availability

The dataset used and/or analyzed during the current study are available from the corresponding author on reasonable request.

## Igbo language version of the 15-item Geriatric Depression Scale

Biko ghọrọ ụsa kachasị kọwaa udị o si metụta gị n'ime izu ụka gara aga.

1. O pekata mpe, Ị nwere afọ ojuju n'ebe ndụ gị dị? **Ee**/**mba**
2. Ị hapụgo ọtụtụ ihe ndị ị na-eme ya na ihe ndị na-amasị gị? **Ee**/**mba**
3. Ọ na-adị gị ka ndụ gị atọgbọziri chakoo? **Ee**/ **mba**
4. Ị naghị enwe uchu ịme ihe ọ bụla? **Ee**/**mba**
5. Ị na-enwekarị obi sara sara? **Ee**/**mba**
6. Ị na-atụ egwu na ihe ọjọọ ga-eme gị? **Ee**/**mba**
7. Ị na-enwe obi aṅụrị ọtụtụ oge? **Ee**/**mba**
8. Ị na-enwekarị nkoropu obi? **Ee**/**mba**
9. Ị na-enwe mmasị ị nọ n'ụlọ karịa ị pụ apụ na ị megasị ihe ọhụrụ? **Ee**/**mba**
10. Ọ na-adị gị ka ị nwekarịrị nsogbu ịcheta ihe karịa ọtụtụ ndị ọzọ? **Ee**/**mba**
11. Ị na-eche na ọ bụ ihe magburu onwe ya na ị dị ndụ ugbu a? **Ee**/**mba**
12. Ọ na-adị gị ka na ị baghịzị uru n'udị ị dị ugbu a? **Ee**/**mba**
13. Ọ na-adị gị ka íke e juru gị ahụ? **Ee**/**mba**
14. Ọ dị gị ka ọnọdụ gị enweghị olileanya? **Ee**/**mba**
15. Ị na-eche na ọtụtụ mmadụ ka gị mma? **Ee**/**mba**

## Abbreviations

BT1: The first back-translated version of the Geriatric Depression Scale
BT2: The second back-translated version of the Geriatric Depression Scale
E-GDS: English version of the Geriatric Depression Scale
I-GDS: Igbo version of the Geriatric Depression Scale
MDD: Minimal detectable difference
PCA: Principal Component Analysis
GDS: Geriatric Depression Scale
SEM: Standard error of mean
T1: First Igbo translation of the Geriatric Depression Scale
T2: Second Igbo translation of the Geriatric Depression Scale
T12: The synthesized or common Igbo translation of the Geriatric Depression Scale
